# Cell-pattern disordering during convergent extension in *Drosophila*

**DOI:** 10.1088/0953-8984/16/44/005

**Published:** 2004-10-22

**Authors:** Jennifer A Zallen, Richard Zallen

**Affiliations:** 1 Department of Molecular Biology, Princeton University, Princeton, NJ 08544, USA; 2 Department of Physics, Virginia Tech, Blacksburg, VA 24061, USA

## Abstract

Convergent extension is the cell-rearrangement process by which a developing embryo elongates to establish the head-to-tail body axis. In the early *Drosophila* embryo, this process occurs within a one-cell-thick epithelial layer. Using confocal microscopy, images were collected of the two-dimensional cell pattern at four stages during convergent extension in wild-type embryos and at one stage in two classes of mutant embryos. The cellular topology was analyzed in terms of the statistical distribution p(n), the frequency of occurrence of n-sided cells. For wild-type embryos, the results demonstrate progressive cell-pattern disordering during convergent extension. The second moment (variance) of p(n) triples to 1.1 while the peak at p(6) drops from 0.65 to 0.38. The fraction of fourfold vertices (four edges meeting) increases from 2% to 8%. Quantitative analysis of interface orientations reveals that the initial degree of hexatic edge-orientational order essentially disappears during the course of convergent extension. The degree of cell-pattern disordering in the two mutants resembles distinct stages in the wild type and correlates with the extent of axis elongation.

## 1. Introduction

Convergent extension is the cell-rearrangement process by which a developing embryo elongates to establish the anterior–posterior (A–P, head-to-tail) body axis in both vertebrates and invertebrates [1, 2]. In *Drosophila*, this multicellular reorganization can occur without cell division [3] or the appearance of significant cell-shape anisotropy [4]. Nearest-neighbor cells in the A–P direction become separated by the intercalation of cells from adjacent rows, producing extension along the A–P axis and narrowing (convergence) along the transverse dorsal–ventral (D–V, back-to-front) axis [4]. Recent studies of *Drosophila* have demonstrated a preferential enrichment of specific proteins at cell–cell interfaces perpendicular to the A–P axis (nonmuscle myosin II) and the D–V axis (Bazooka/PAR-3), providing insight into the mechanism for cell rearrangement during convergent extension [5–7].

Convergent extension in *Drosophila*, also known as germband extension, occurs within a one-cell-thick epithelial layer. Confocal microscopy of the two-dimensional cell-packing pattern within this layer suggests that the cell pattern becomes progressively less ordered as convergent extension proceeds. Disordered cellular patterns have a long history of scientific study in various contexts, such as grain shapes in polycrystalline solids, soap bubbles in foams, and atomic cells in amorphous solids. Recent reviews can be found in the book by Weaire and Hutzler [8] and the review by Schliecker [9]; the pioneering earlier article by Weaire and Rivier [10] remains a useful source. Quantitative characterizations of disordered cellular structures are therefore available. In this paper, we use several approaches to obtain quantitative measures of the changing cell-pattern topology during convergent extension in *Drosophila*. The cell-pattern images were obtained using a Zeiss LSM 510 laser-scanning confocal microscope. Embryonic genotypes were Oregon R (wild type), *eve*^R13^ homozygotes (denoted *eve* mutant) and *kni* ^IID48^ *hb*^7M48^ *fkh*^E200^ *tll*^L10^ homozygotes (*khft* mutant). Mutant embryos were identified by altered Eve expression. Cell surfaces were visualized in fixed embryos using fluorescence-conjugated phalloidin or antibodies to the Armadillo and Neurotactin proteins, as described in [5].

## 2. Cell-topology distributions

**Figure 1.**
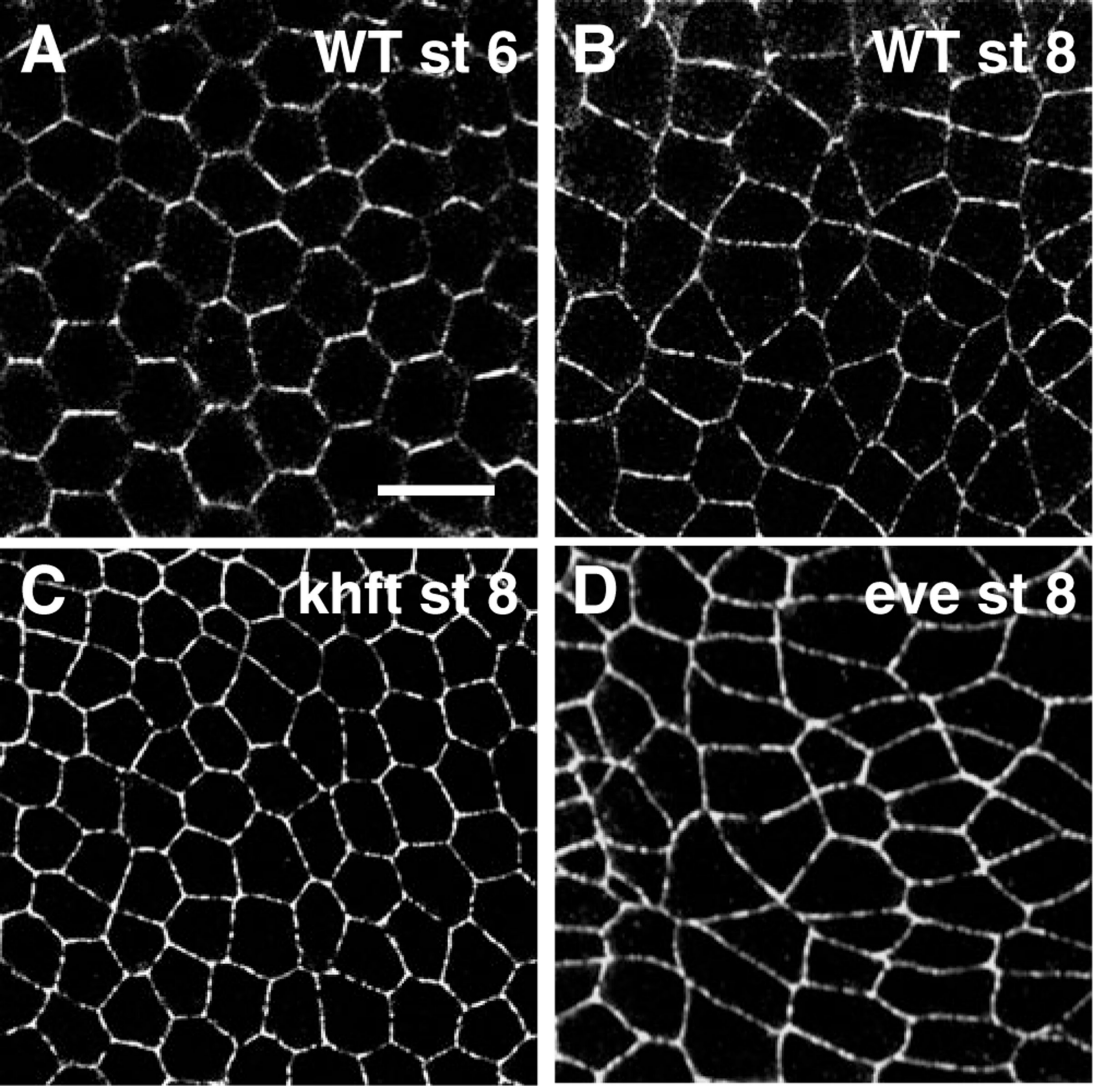
Confocal images of the germband cell layer in early *Drosophila* embryos. (A) Wild-type stage-6 embryo, just prior to the onset of convergent extension. (B) Wild-type stage-8 embryo, in the process of convergent extension. (C) *khft* mutant embryo at stage 8. (D) *eve* mutant embryo at stage 8. Anterior is the left and dorsal is up; all images were taken in the anterior region of the germband. Scale bar = 10 *μ*m. Each image represents a 1.2 *μ*m-thick optical slice.

Figure 1 displays two images taken of wild-type (normal) embryos and two taken of mutant embryos lacking specific gene products. Stages 6 and 8 refer to the well characterized organism-scale morphological stages of embryo development introduced by Wieschaus and Nusslein-Volhard [11]. Stage 6 is just prior to the onset of convergent extension, while stage 8 is at the time of intercalation. The square field of each micrograph image is 46 *μ*m on an edge.

**Figure 2.**
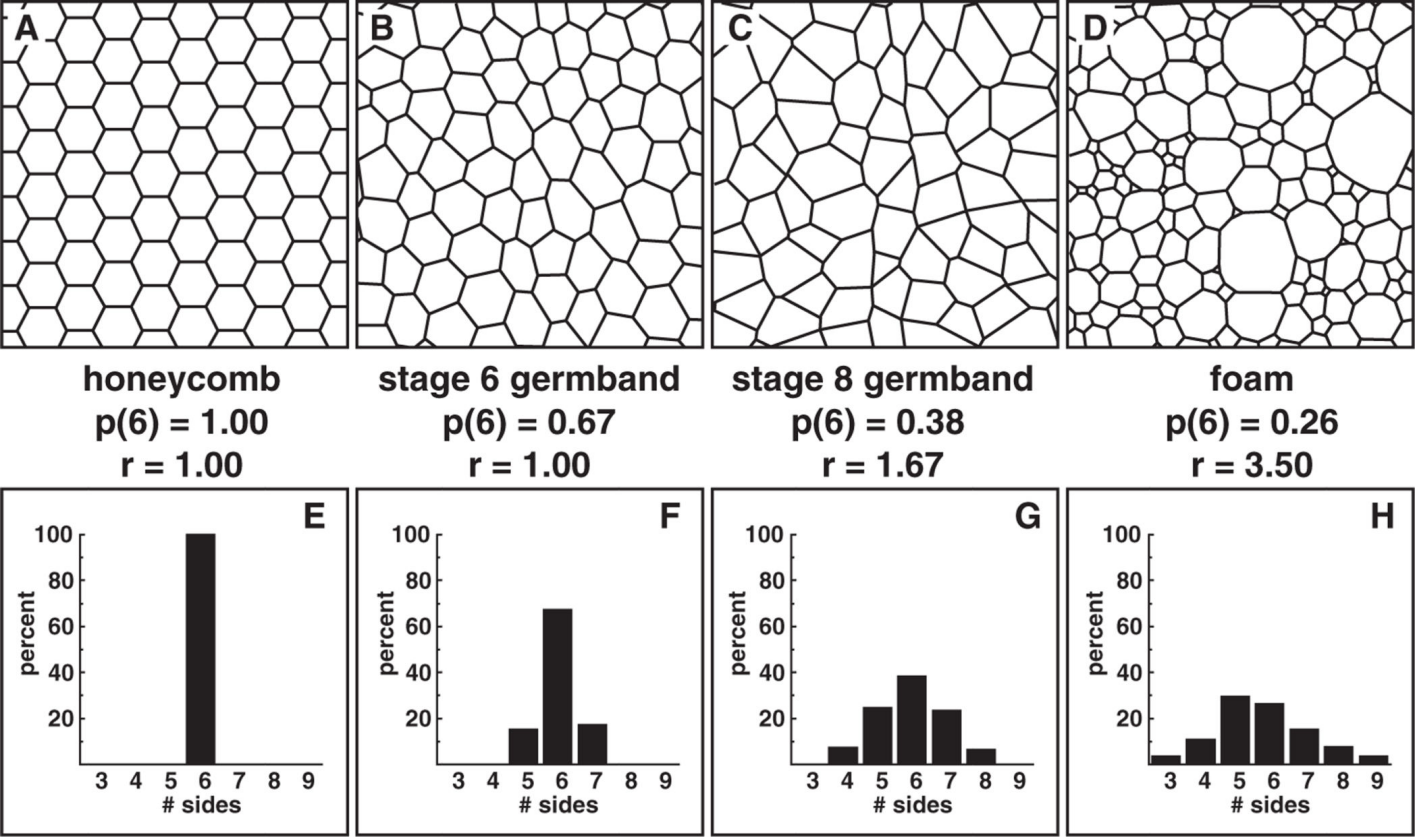
Comparison of two wild-type cell patterns with the perfectly ordered honeycomb and a highly disordered foam. The histograms show the p(n) polygon statistics. The fraction of six-sided cells, p(6), and the ratio, r, is provided for each pattern. These images fall along a continuum of disorder: the wild-type stage-6 embryo more closely resembles the honeycomb and the wild-type stage-8 embryo displays increased disorder characteristic of the foam.

To place these structures on a rough scale representing the degree of disorder, Figure 2 contains a comparison with two extreme examples: the perfectly ordered honeycomb lattice (Figure 2A) and a highly disordered foam (Figure 2D). The foam shown was observed as a two-dimensional soap froth confined between glass plates [10, 12]. Shown below each cell pattern is the corresponding cell-topology distribution p(n), where p(n) is the fraction of cells having n sides. The honeycomb consists entirely of hexagons: p(6)=1 and the distribution is perfectly sharp. With increasing disorder, the distribution broadens and p(6) drops.

To quantify changes in cell pattern during convergent extension in the *Drosophila* embryo, the cellular topology was examined for 43 such images. These images included six different cases: wild-type embryos at stages 6, 7, 8, and late stage 8 (approximately 10, 20, 30, and 40 min, respectively, after the formation of the epithelial cell layer by cellularization [11]), and two mutants (*khft* and *eve*) at stage 8. Seven images were studied for each case, except for wild type at late stage 8 (eight images). The number of sides for each cell was recorded, counting only cells that were wholly within the field. The average number of cells per image was 43, with over 1800 cells counted in all.

For two-dimensional cell patterns with three edges meeting at each vertex z=3, the average number of sides 〈n〉 is necessarily equal to six [8–10]. While a small minority of fourfold vertices (discussed in section 3) are observed in the germband cell patterns, the p(n) distribution was determined by treating the occasional z=4 vertex as a pair of unresolved adjacent z=3 vertices. The assumed short edge separating the unresolved vertices could occur in two possible ways (see the discussion of the T1 process in section 3). To account for this, a cell with five observed sides and one fourfold vertex was counted as contributing 0.5 to the number of n=5 cells and 0.5 to the number of n=6 cells. For our full data set, 〈n〉 was 5.98. In estimating the variance, the second moment of (n−〈n〉), we used 〈n〉=6.

**Figure 3.**
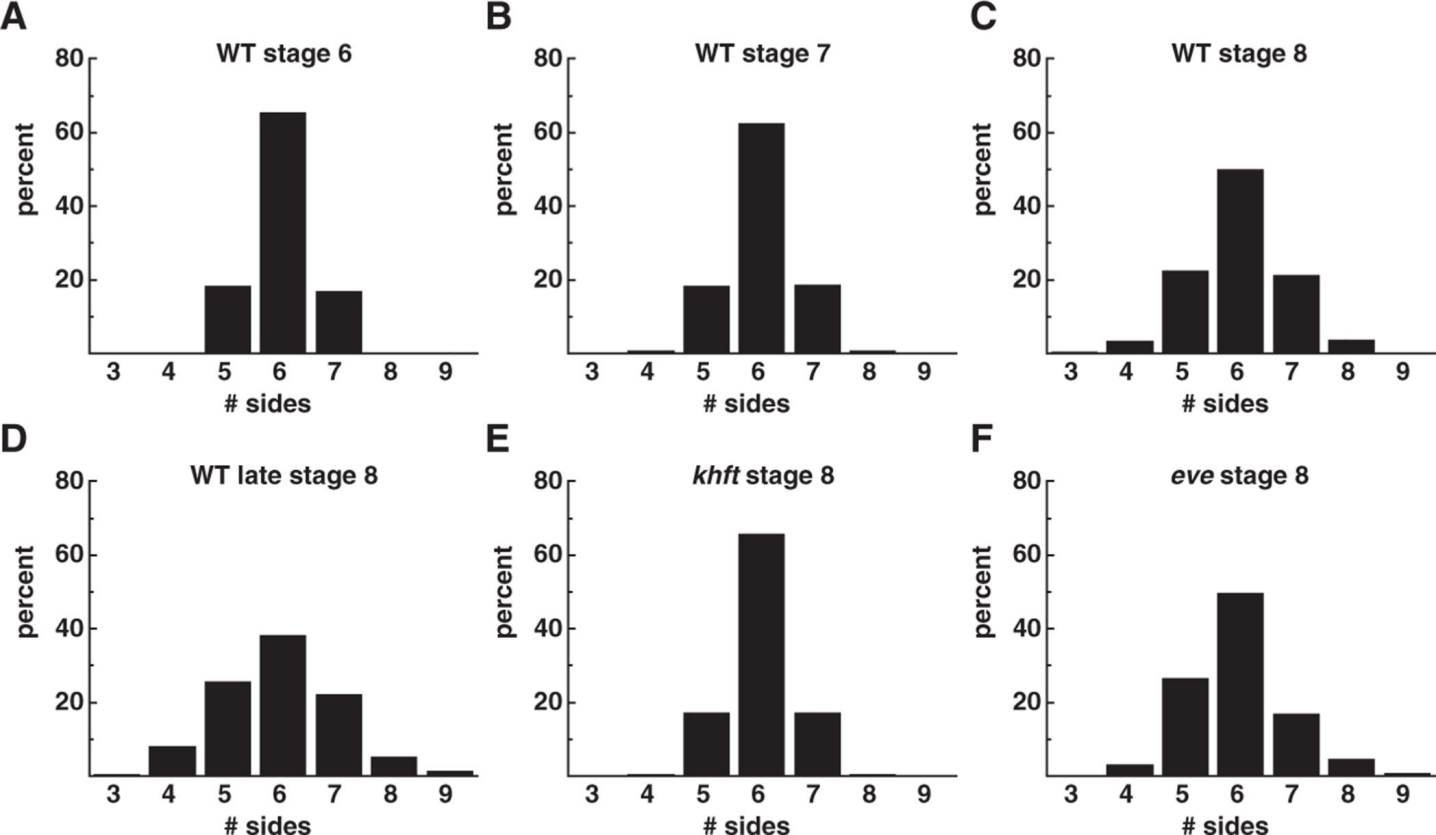
Observed cell-topology distributions for six classes of *Drosophila* embryos investigated. (A)–(D) Wild-type embryos at stages 6, 7, 8, and late 8 (corresponding to 10, 20, 30, and 40 min after the formation of the germband cell layer, respectively). (E) *khft* mutant at stage 8. (F) *eve* mutant at stage 8.

The average distributions of p(n) values for each case are shown in Figure 3. In wild-type *Drosophila* embryos at stage 6, prior to the onset of intercalation, the majority of cells are hexagonal with a minority of five- and seven-sided cells (Figures 2B, 3A). During the period of cell intercalation in stage-8 embryos ( ~20 min later), the fraction of hexagonal cells decreases to a minority, with an increase in the number of pentagonal and heptagonal cells (Figures 2C, 3C). Moreover, cells with four and eight sides appear at stage 8 that are never observed in stage-6 embryos. The fraction of nonhexagonal cells continues to increase as convergent extension continues in late stage 8 (Figure 3D).

Patterned gene expression along the A–P axis is required for convergent extension in the *Drosophila* embryo [4, 5]. This A–P pattern is eliminated in the germband of *khft* mutants and no cell intercalation occurs [4]. Consistent with this, *khft* mutants also fail to acquire the cell-pattern disorder characteristic of wild-type stage-8 embryos (Figure 3E). In contrast, some degree of A–P pattern is retained in *eve* mutant embryos, and these embryos acquire a significant amount of topological disorder (Figure 3F). Accordingly, the body axis undergoes partial elongation in *eve* mutants [4].

**Figure 4.**
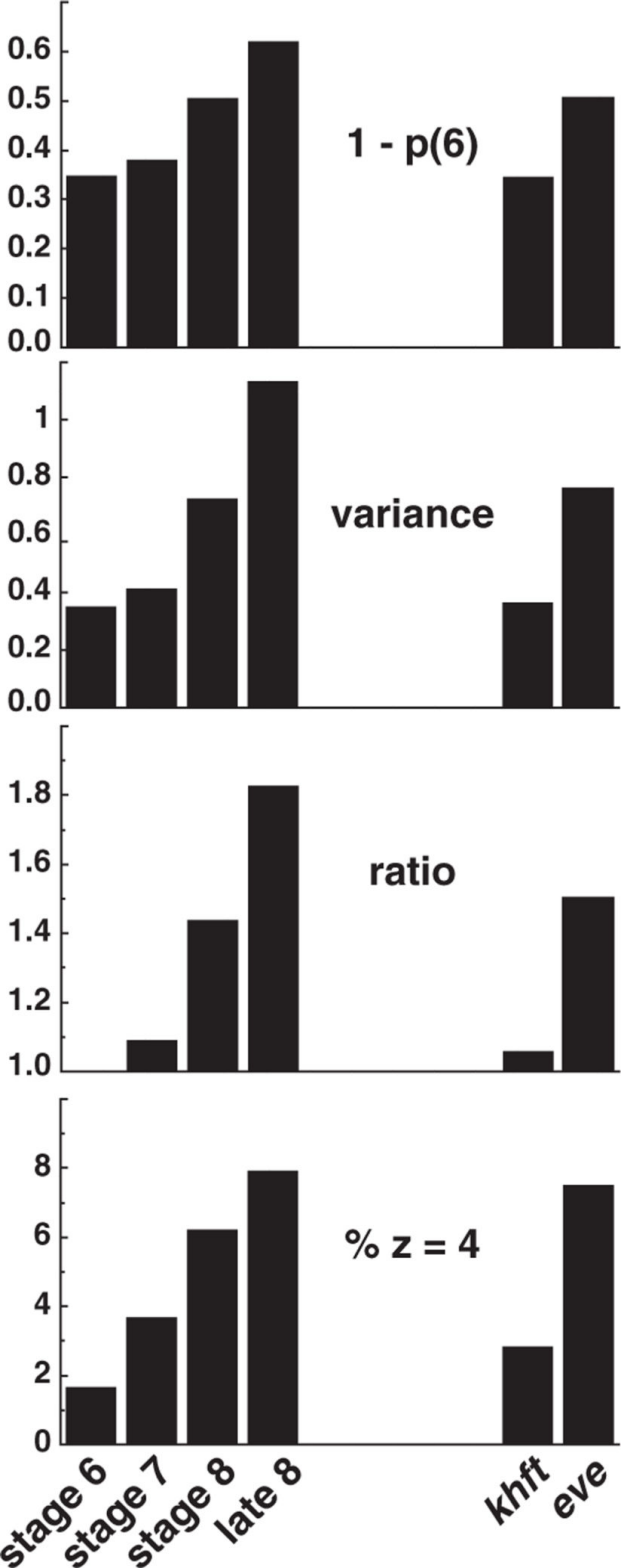
Four quantitative measures of cell-pattern disorder for six classes of *Drosophila* embryos: the fraction of nonhexagonal cells, the variance of p(n), the ratio r discussed in the text, and the fraction of fourfold vertices. The four classes of embryos plotted on the left are wild-type embryos at progressive stages of convergent extension. The two classes on the right are *khft* and *eve* mutants at stage 8.

Both the fraction of nonhexagonal cells, 1−p(6), and the variance of the distribution are measures of the degree of disorder in the cell pattern. Our results for these two quantities in the six classes of embryos are summarized in Figure 4. Also included is the ratio r discussed by Schliecker [9], defined as the variance divided by [1−p(6)]. This quantity is noteworthy here because it is equal to unity when only five-, six-, and seven-sided cells are present, which is the situation observed for stage-6 wild-type embryos (Figure 3A). In this situation, the variance is equal to p(5)+p(7) which, in turn, equals [1−p(6)]. As disorder increases and the p(n) distribution broadens, r increases above unity.

In wild-type embryos, all three p(n)-distribution quantities plotted in the top three panels of Figure 4 show the same systematic trend: the cell topology becomes progressively more disordered during convergent extension. For the two types of mutant embryos, the degree of disorder acquired at stage 8 correlates well, when compared to wild type, with the degree of convergent extension. In *khft* mutants where no convergent extension occurs, the cell topology resembles that of stage-6 wild-type embryos prior to the onset of cell rearrangement. In contrast, some degree of intercalation does occur in *eve* mutant embryos, and *eve* mutants acquire the topological disorder characteristic of wild-type stage-8 embryos engaged in cell rearrangement.

## 3. T1-process fourfold vertices

While the great majority of vertices (edge intersections) in the images are threefold vertices, some fourfold vertices are observed and their frequency of occurrence increases as convergent extension proceeds. At a fourfold (z=4) vertex, four cells (as well as four edges) meet at one point in the two-dimensional cell pattern. A z=4 vertex automatically appears as a transient during a ‘T1 process’, the fundamental neighbor-switching topological rearrangement in two dimensions first elucidated by Weaire and co-workers in 1983 [10, 13]. This process has recently been used to describe cell rearrangement during convergent extension in *Drosophila* [6]. Let A/B denote the edge between cells A and B. Consider a cluster of four cells containing A/B, B/C, C/D, D/A, and A/C interfaces. B and D do not touch but are separated by cells A and C and the length of the A/C edge. If the A/C edge shrinks in length to zero, two z=3 vertices merge into one z=4 vertex where all four cells meet. To complete the T1 neighbor switch, a B/D edge forms so that B and D are now neighbors while A and C no longer are. The cell arrangement topologically changes in a T1 process, and two cells (here, B and D) each gain an edge while two cells (C and D) lose an edge. The total number of cells, edges, and vertices does not change.

Since a z=4 vertex can be viewed as a neighbor-switching cell rearrangement caught in the act, we recorded the number of z=4 vertices observed in our micrographs (Figure 4, bottom panel). For each image, the frequency of fourfold vertices was obtained as the fraction of z=4 vertices relative to the total number of vertices contained in the field. In wild-type embryos, the fourfold-vertex fraction increases from under 2% in stage 6 to 8% in late stage 8. This measure follows the same trend as the other cell-pattern disorder measures shown in Figure 4.

Bertet and colleagues also note an increasing number of fourfold vertices (which they refer to as type-2 junctions) during convergent extension in *Drosophila* [6]. Since they present their results in terms of a ratio involving the number of edges oriented in certain directions (angular range unspecified), we cannot directly compare their results to ours. However, their measurements also indicate an increase in z=4 vertices by about a factor of four during convergent extension, so the two sets of experiments are in general agreement.

## 4. Edge-orientational disorder

**Figure 5.**
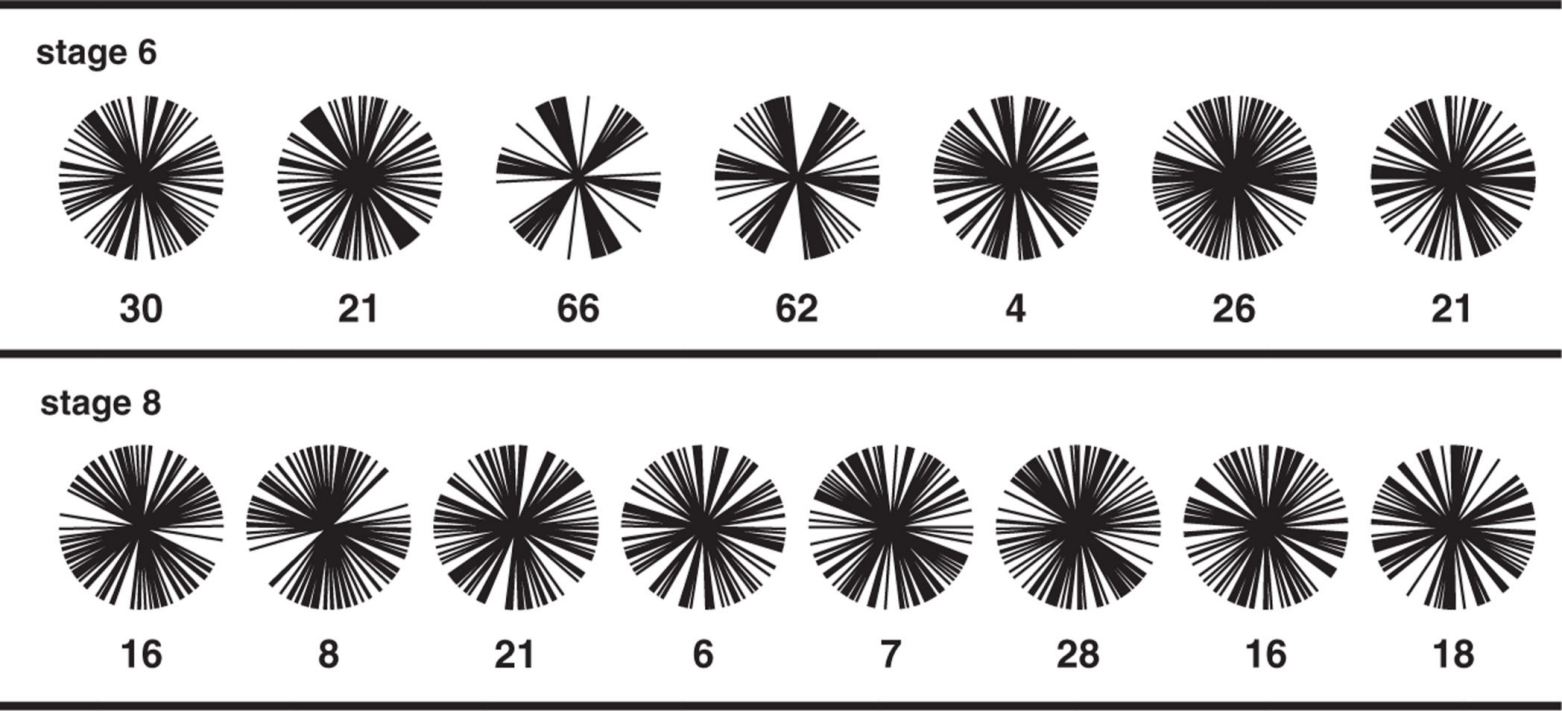
Interface orientations for stage-6 and stage-8 wild-type embryos. Sixty interfaces were measured for each image. The number below each orientation-distribution plot is 100$Q_{6}$, where $Q_{6}$ is the hexatic edge-orientational order parameter determined from the distribution.

The honeycomb structure (Figure 2A) has complete edge-orientational order; one-third of the edges are horizontal (0^◦^), one-third are at 60^◦^, and one-third are at 120^◦^. In stage 6 in the developing *Drosophila* embryo, while hexagons are in the majority in the germband pattern, they are irregular (Figure 2B) and coexist with a substantial minority (35%) of five-sided and seven-sided cells (Figure 3A). These results raise the question of whether there is significant edge-orientational order in this structure. We measured edge orientations for 60 interfaces in each of the 15 images taken of stage-6 and stage-8 wild-type embryos (Figure 5).

An interface oriented close to the vertical, having a near-horizontal normal that is approximately parallel to the A–P axis, is referred to as an A–P interface. In our analysis we consider interfaces oriented within 18^◦^ of vertical to be A–P interfaces and interfaces oriented within 18^◦^ of horizontal to be D–V interfaces. Each of these angular ranges comprises 20% of the full range. For 420 interfaces measured in seven wild-type stage-6 embryos, 22% were A–P interfaces and 25% were D–V interfaces. For 480 interfaces measured in eight wild-type stage-8 embryos, 25% were A–P and 18% were D–V. No pronounced orientational preference is observed. A recent model for axis elongation in *Drosophila* proposes that A–P interfaces are progressively replaced by D–V interfaces [6]. Our interface-orientation results do not provide evidence for such a trend, indicating that convergent extension does not involve a systematic transition from A–P to D–V interfaces.

The quantity $Q_{6}$, used to provide a measure of hexatic bond-orientational order in two- dimensional structures [14], is also useful to consider for cell patterns in the *Drosophila* embryo. For a sample consisting of N edges, $N\;Q_{6}$ is defined as the absolute magnitude of the sum over edges of exp(i6θ), where θ is the angle of each interface relative to a fixed reference axis. $Q_{6}$ is independent of the choice of reference axis. The square of $N\;Q_{6}$ is equal to the square of the sum of cos(6θ) for all interfaces in each image plus the square of the sum of sin(6θ) for all interfaces. The factor of six ensures that interfaces differing by 60^◦^ contribute constructively to the sums. Randomly oriented interfaces tend to cancel each other out. Thus $Q_{6}$ is 1.00 for the honeycomb and zero for randomly oriented edges in the large-N limit. For orientational correlations extending over a characteristic correlation length, $Q_{6}$ will fall off with increasing sample size. We used a sample size of 60 edges in each image, contained within a 46 *μ*m × 46 *μ*m region representing ~10% of the germband. For each sample, the corresponding $Q_{6}$ value is indicated in Figure 5.

In stage-6 embryos, the $Q_{6}$ distribution has a mean value of 0.33 and a standard deviation of 0.21. Both the mean and six of the seven individual values are well above 0.13, the random-walk value (reciprocal of the square root of 60) for N=60. Thus, in stage 6, orientational correlations extend over a region containing 60 edges. For the two images with $Q_{6}$ above 0.6, the orientational order is readily apparent (Figure 5). In stage-8 embryos at the time of convergent extension, the $Q_{6}$ distribution has a mean value of 0.15 and a standard deviation of 0.07. The mean is not significantly different from 0.13, the result expected in the absence of orientational order. We determined the effect of weighting the orientations by the edge lengths, and found the effect on $Q_{6}$ to be small.

These results demonstrate that edge-orientational disorder in the cell pattern increases substantially during convergent extension in the *Drosophila* embryo, with very little orientational order remaining at stage 8, the period of sustained intercalation. Over a region corresponding to ~10% of the germband layer, the hexatic orientational order is modest in stage 6 and is essentially absent in stage 8. At stage 8, $Q_{6}$ is indistinguishable from the random-walk value.

## 5. Summary

We have investigated the cell-pattern topology of *Drosophila* embryos during convergent extension, the multicellular reorganization of the germband cell layer that results in elongation of the body axis. Quantitative results obtained for the p(n) distribution (Figure 3) and statistical measures for this distribution (Figure 4) demonstrate progressive cell-pattern disordering during this process. Analysis of interface orientations (Figure 5) shows no evidence of a strongly preferred interface orientation. Modest hexatic orientational order is present in stage 6 and essentially disappears by stage 8. Interestingly, two classes of mutant embryos display distinct levels of disorder during the period of cell rearrangement in stage 8. *khft* mutants that fail to undergo convergent extension retain the ordered cell pattern present in stage-6 wild-type embryos, while *eve* mutants that carry out partial cell rearrangement exhibit a cellular disordering similar to stage-8 wild-type embryos. Thus the degree of cell-pattern disordering correlates with the extent of axis elongation in these mutants. These observations combine developmental biology with physics techniques to generate new measures for cell rearrangement that are distinct from previous methods based on axis elongation or intercalary cell behavior. Our analysis reveals that the organized cell rearrangements that elongate the body axis during convergent extension in *Drosophila* are accompanied by an increase in disorder on the cellular level.
